# Reviewer’s comment concerning “3D analysis of brace treatment in idiopathic scoliosis” (ESJO-D-12-00300R” by A. Courvoisier, X. Drevelle, R. Vialle, J. Dubousset and W. Skalli)

**DOI:** 10.1007/s00586-013-2969-0

**Published:** 2013-08-27

**Authors:** Joseph Y. Margulies

**Affiliations:** Spine Surgery, 8 Usonia Road, Pleasantville, NY 10570 USA

As we all know, “idiopathic” as in “idiopathic scoliosis” means in plain English “without apparent cause”, and practically “without a cause that we know”. This also means that our repertoire of treatment of this “idiopathic scoliosis” is symptomatic at the most, unlike a definitive treatment given using braces in some non-idiopathic types of scoliosis, for example, paralytic scoliosis.

Many times symptomatic treatment is practically asymptotic and dangerously close to the basic rule of “primum no nocere”, if not crossing it. “Idiopathic scoliosis” is actually not a nosologic entity on its own, but rather a term to generally classify a common symptom of other diverse phenomena, not yet sorted out and not yet separately defined and classified. As of today there are over 150 syndromes that include scoliosis of a kind as one of their symptoms; in some the scoliosis is the major and central symptom. In some of them, the pathological process is very clear and the biomechanical consequences well figured out and hence directly treatable: once the cause is clear the road for specific definitive treatment is open; and this includes the use of braces as a definitive treatment for specific patterns of scoliosis in specific diseases.

However braces, as all other treatment modes of “idiopathic scoliosis” including surgical correction and fusion, are only a mode of symptomatic treatment. What we call “idiopathic scoliosis” is most probably a large collection of different nosologic entities. As mentioned in the paper above, the success of treatment using braces varies widely. This is not surprising considering the fact that braces are inadvertently used to treat a whole slew of different nosologic entities (though not yet defined…). The success is rather a result of a trial and error process and of sheer luck.

Braces in the regime of treatment in “idiopathic scoliosis”, however, can be utilized as an important tool of research in the quest for the cause of curves in various “scolioses”. Rather than using them as treatment, they can be used as therapeutic trials and considered as a sorting tool to identify possible specific groups among the “idiopathic scoliosis” population. We can make an attempt to identify a group of patients who reacted positively and were improved by the braces and are otherwise similar in their age group, gender, specific mechanical and structural curve characteristics (like levels, angels and flexibility), etc. Then we may be able to identify further structural or biological elements in this specific group and define a new nosologic entity external to the “idiopathic scoliosis” population.

The measuring tool described in the paper above is a very advanced and sophisticated method for the purpose of sorting various structural characteristics of curves. Moreover, ranges of motion can be measured in all axes, having the patient documented going through all the “cone” of range of motion of the spine, hopefully in a standing position. We do have to assume that even curves presenting with the same static characteristic may have totally different ranges of motion and may belong to different nosologic entities; they still may have different initiating and deforming factors and represent different diseases.

Please note that curve patterns and classifications are specifically not mentioned here. It is fair to say that all existing structure-based classifications are aimed at guiding the treatment of the deformity mechanically rather than etiologically (and occasionally to bury the deformity under a heavy “sarcophagus” of fusion mass). In other words, the existing structural based classifications of idiopathic scoliosis seem to serve treatment strategies rather than etiological sorting.

The word “idiopathic” added to the “*Keywords: scoliosis; brace; 3D; variability; stereoradiography*”, used in the above paper, can be used in a different future paper: a study structured in a prospective manner, aiming at discovering an etiology in a selective and very homogeneous group of patients who also responded positively to uniformly designed brace treatment; all this using the very modern high-power spine imaging post-processing techniques.

